# Oral health in patients with severe inflammatory dermatologic and rheumatologic disease

**DOI:** 10.1002/ski2.156

**Published:** 2022-08-07

**Authors:** Yvonne Kiernan, Cathal O’Connor, John Ryan, Michelle Murphy

**Affiliations:** ^1^ Department of Medicine University College Cork Cork Ireland; ^2^ Department of Dermatology South Infirmary Victoria University Hospital Cork Ireland; ^3^ Department of Rheumatology Cork University Hospital Cork Ireland

## Abstract

**Background:**

Poor oral health (OH) is a risk factor for systemic disease and lower quality of life (QoL). Patients with inflammatory dermatologic/rheumatologic diseases report more oral discomfort, dry mouth, and periodontal disease than controls. Medications used to treat these conditions can also adversely affect OH.

**Objectives:**

The aim was to assess the OH of patients with chronic inflammatory dermatologic/rheumatologic diseases treated with systemic/biologic therapy, compared to controls.

**Methods:**

Patients with chronic inflammatory dermatologic/rheumatologic diseases treated with systemic/biologic therapy were recruited from outpatient clinics across two university hospitals. All patients had a standardized World Health Organisation OH assessment performed consisting of an OH exam and questionnaire. Age‐ and sex‐matched controls without chronic inflammatory disease were recruited from a pigmented lesion clinic. Charts of patients with chronic inflammatory dermatologic/rheumatologic diseases were reviewed to assess OH documentation.

**Results:**

One hundred patients were examined (50 cases and 50 controls). Patients with inflammatory dermatologic/rheumatologic diseases (cases) had poorer periodontal status (mean loss of attachment 6.9 mm vs. 1.9 mm controls, *p* = 0.01), more missing teeth (mean 7.7 vs. 4.4 controls, *p* = 0.029), more dry mouth (82% vs. 20% controls, *p* = 0.001), and less frequent tooth brushing (60% vs. 80% controls, *p* = 0.037). Of 250 patient charts which were reviewed, 98.4% (*n* = 246) had no documentation of OH.

**Conclusion:**

Patients with severe inflammatory dermatologic/rheumatologic conditions have poorer OH and OH‐related QoL. Clinicians should appreciate the risk of poor OH in this cohort and have a low threshold for involving OH professionals in care pathways for severe inflammatory disease.

## Graphical abstract

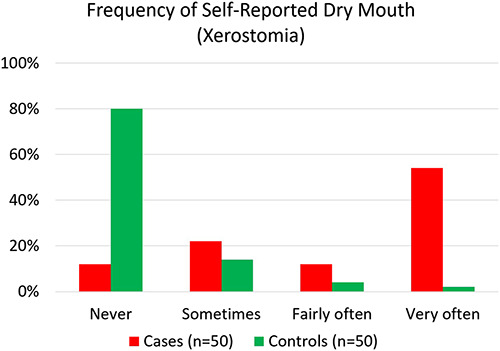

Poor oral health has a bidirectional relationship with immune‒mediated skin and joint disease. In this study, patients with severe inflammatory rheumatological and dermatological diseases had worse periodontal disease, more missing teeth, more dry mouth, less frequent tooth brushing, and poorer quality of life. Clinicians should be aware of this relationship, screen for poor oral health, and have a low threshold for involving oral health professionals in caring for patients with severe inflammatory skin and joint disease.

1

**What is already known about this topic?**

- Poor oral health (OH) is a risk factor for systemic disease and lower quality of life (QoL).
- Patients with inflammatory rheumatologic and dermatologic diseases are at increased risk of poor OH due to disease activity, maladaptive behaviours such as smoking or alcohol ingestion, and medications used to treat their immune‐mediated disease.
- Patients on immunomodulatory therapy may benefit from OH screening.

**What does this study add?**

- Patients with severe inflammatory dermatologic and rheumatologic conditions have poor periodontal health, more missing teeth, more dry mouth, less tooth brushing, and poor OH related QoL.
- Dermatologists and rheumatologists should be aware of the risk of poor OH in this cohort, have a low threshold for screening for oral disease, and involve OH professionals in care pathways for severe inflammatory disease.

## 2 INTRODUCTION

The World Health Organisation (WHO) defines oral health (OH) as a state of being free from “oral and facial pain, oral and throat cancer, oral infection, periodontal disease, tooth decay, tooth loss, and other diseases and disorders that limit an individual's capacity in biting, chewing, smiling, speaking, and psychosocial wellbeing”.^1^ OH is essential to general health and QoL,^2^ and the impact of poor OH on health and QoL is increasingly recognised. Poor OH has been associated with adverse pregnancy outcomes,^3^ cardiovascular disease,^4^, ^5^ pulmonary disease,^6^ and diabetes,^7^ although causal relations have not yet been clearly established.^8^ Patients with inflammatory dermatologic and rheumatologic diseases have reported more oral discomfort/pain, higher levels of periodontal disease, dry mouth and oral mucosal lesions (OML) than healthy controls.^5^, ^6^ In addition, while many medications are reported to cause dry mouth,^9^ systemic medications used to treat these conditions can cause adverse oral side effects such as dry mouth and mucositis, which can impact negatively on maintenance of OH.^8^

Psoriasis has been linked with a higher prevalence of oral lesions such as geographic tongue, fissured tongue and oral candidiasis compared to control subjects.^10^, ^11^ Higher rates of periodontitis have also been described in psoriasis.^12^, ^13^ Rheumatoid arthritis (RA) has been associated with an increased frequency of desquamative gingivitis and periodontal disease.^14^, ^15^ A bidirectional relationship between periodontitis and RA has been proposed, in which people with RA are more likely to develop periodontitis,^16^ and periodontitis may play a role in the initiation or maintenance of systemic inflammation and autoimmune response.^17^ Patients with systemic lupus erythematosus frequently have oral ulcers, periodontal disease, and temporomandibular join (TMJ) dysfunction, with severe impact on QoL.^18^ Salivary exocrine insufficiency in Sjogren's syndrome (SS) results in hyposalivation that predisposes to OH complications such as dental caries, periodontal disease, and difficulty eating or wearing dentures,^19^ with significant impact on QoL.^20^, ^21^ Both RA and ankylosing spondylitis are associated with TMJ inflammation and dysfunction.^22^ Poor OH has also been observed in patients with Behcet's disease and is associated with a more severe disease activity.^23^

Oral and biologic immunomodulators are used extensively to treat inflammatory dermatologic and rheumatologic diseases, but can result in adverse oral side effects, such as oral ulceration, mucositis and dry mouth, which can impact on OH.^8^ Furthermore, the potential association between immunosuppressive therapy and oral cancer^24^, ^25^ means that patients on systemic or biologic agents may benefit from regular OH review. Systemic treatment involves the use of medication in the form of tablets of injections. Biologic treatment specifically refers to treatment using substances made from living organisms to treat disease, such as monoclonal antibodies.

The aim of this study was to prospectively assess the OH and OH‐related QoL in patients with a chronic inflammatory dermatologic or rheumatologic condition treated with systemic or biologic therapy, compared to controls.

## 3 MATERIALS AND METHODS

This was a prospective observational cross‐sectional study with two groups – cases (chronic inflammatory dermatologic or rheumatologic disease) and controls (no history of chronic inflammation).

### 3.1 Study groups

The Clinical Research Ethics Committee of the Cork Teaching Hospitals approved the study protocol. Informed consent was obtained from all participating subjects prospectively and prior to invitation for the dental examination. Case inclusion criteria were adults attending dermatology or rheumatology outpatient clinics with a consultant‐diagnosed chronic inflammatory disease, treated with systemic or biologic therapy. Children (under 18 years) and pregnant women were excluded. Cases were recruited from dermatology and rheumatology outpatient clinics in Cork University Hospital and the South Infirmary Victoria University Hospital (SIVUH) Cork, Ireland. Age‐ and sex‐matched controls, with no history of inflammatory systemic disease, were recruited from a pigmented lesion clinic in SIVUH.

Participants were divided into binary groups according to whether they

(a) Had never smoked or had not smoked in over 12 months
(b) Were current smokers or had smoked in the last 12 months

All consenting patients had an OH assessment. This comprised of an OH questionnaire and a physical OH exam performed by a dentist using internationally standardised templates from the WHO.^1^

### 3.2 Oral health questionnaire

The OH questionnaire (Supporting Information S1) from the WHO^1^ comprises of 16 questions relating to self‐assessed OH, oral pain/discomfort, dry mouth, oral hygiene habits, OH‐related QoL, diet, smoking, alcohol and education. The OH‐related QoL question (question 12) measures functional, psychological, emotional and social disability associated with poor OH. Using a five‐point scale ranging from 4 (very often) to 1 (never), with 0 representing a ‘don't know’ answer, participants rated how frequently they had experienced each symptom during the past 12 months.

### 3.3 Oral health examination

All patients were examined under standardised conditions, in their respective dermatology or rheumatology clinic, by the same dentist, in addition to their standard clinical care. This standardised examination from the WHO^1^ (Supporting Information S2) included dental findings, evaluation of periodontal health and presence of OML.

Dental findings: teeth were assessed visually with a mirror, probe and light source. The number of decayed, missing and filled teeth were recorded (out of 28). Only natural teeth were counted.

Periodontal health: the loss of attachment (LOA) in each sextant of the mouth was measured using a millimetre‐scaled WHO community periodontal index (CPI) periodontal probe. The periodontal LOA is the distance from the cemento‐enamel junction of the tooth to the base of the gingival sulcus.^26^ Periodontal disease is defined as a LOA greater than or equal to 5.5 mm.^26^

Oral mucosal lesions: the oral mucosae were examined for the detection of soft tissue alterations (oral lichen planus, leukoplakia, ulceration, candidiasis, geographic tongue, furry tongue, traumatic keratosis, angular cheilitis etc).

Dentures: The presence of a partial or complete upper/lower denture was recorded.

### 3.4 Oral health documentation

The charts of patients with a chronic inflammatory dermatologic/rheumatologic diagnosis were reviewed retrospectively to audit documentation of OH status. Charts were randomly selected from a database of dermatology and rheumatology patients attending biologic clinics in SIVUH. All charts were manually reviewed for any documentation of OH. The medical charts of the study cases were included in the review for any reference to OH.

### 3.5 Statistical analysis

All statistical data was analysed using SPSS Version 22 statistical software. Categorical variables were compared between the two groups by chi‐square test while continuous variables were compared using independent samples t‐test. A Kruskal‐Wallis test was used to test the difference in more than two means and a Mann‐Whitney *U* test was used to compare the median of ordinal variables between groups. The level of significance was set to *P* < 0.05.

## 4 RESULTS

### 4.1 Study cohort

One hundred patients were enroled in this study (50 cases and 50 controls). The demographic data of the study population are provided in Table 1. Mean age (standard deviation, SD and range) of study cases and controls were 50.3 (15.9, 20–83) and 45.4 (17, 19–83) respectively and each group had the same number of males (*n* = 19) and females (*n* = 31). There were no changes to results following adjustment for the minor discrepancy in mean age between the groups. There was a higher proportion of smokers in the control group (34%) than the case group (14%), and a slightly higher proportion of alcohol drinkers (68%) than the case group (54%). Education attainment was similar between groups.

**TABLE 1:** Main demographic characteristics of the study cohort (*n* = 100)

	Cases (*n* = 50)	Controls (*n* = 50)
Mean age, years	50.3	45.4
Standard deviation, years	+/− 15.9	+/− 17
Female sex, *n* (%)	31 (62)	31 (62)
Smoker, *n* (%)	7 (14)	17 (34)
Alcohol, *n* (%)	27 (54)	34 (68)
Education, *n* (%)		
Primary school	1 (2)	1 (2)
Secondary school	36 (72)	28 (56)
University/postgraduate education	13 (26)	21 (42)

### 4.2 Diagnoses and medications

The dermatologic/rheumatologic diagnoses of the case participants are shown in Table 2. The most common diseases included were psoriasis/psoriatic arthritis with 24 cases (48%) and RA with 18 cases (36%). Medications prescribed at the time of assessment are shown in Table 3. The most commonly prescribed treatments were tumour necrosis factor alpha antagonists (62%) and methotrexate (16%).

**TABLE 2:** Inflammatory dermatologic/rheumatologic diagnoses of case participants (*n* = 50)

	*n*	%
Psoriasis/psoriatic arthritis	24	48
Rheumatoid arthritis	18	36
Systemic lupus erythematosus	3	6
Ankylosing spondylitis	2	4
Sjogren's syndrome	1	2
Hidradenitis suppurtiva	1	2
Behcet's disease	1	2

**TABLE 3:** Currently prescribed systemic/biologic agents for case participants (*n* = 50)

	*n*	%
Adalimumab	14	28
Etanercept	14	28
Methotrexate	8	16
Sulfasalazine	3	6
Ustekinumab	2	4
Golimumab	2	4
Hydroxychloroquine	2	4
Infliximab	1	2
Tocilizumab	1	2
Mycophenolic acid	1	2
Leflunomide	1	2
Glucocorticoids	1	2

### 4.3 Oral health questionnaire

**FIGURE 1:**
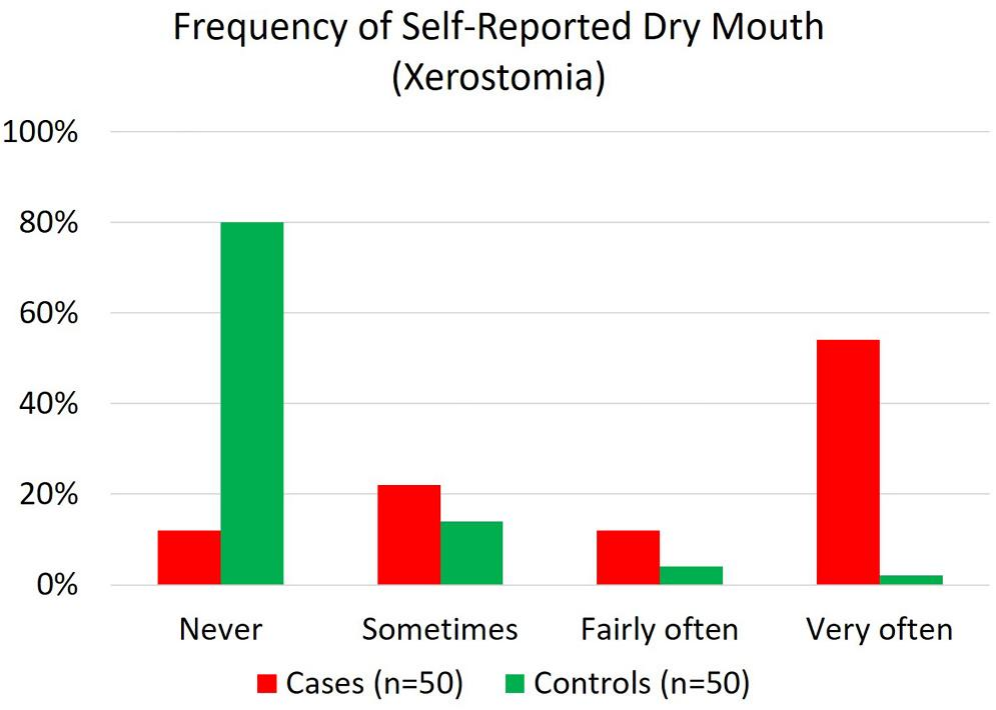
Frequency of dry mouth from oral health (OH) related quality of life (QoL) measures component of OH questionnaire

Results from the OH questionnaire are summarised in Table 4. There was a statistically significant difference in the prevalence of dry mouth reported between cases and controls [82% of cases v 20% controls (*P* = 0.001)]. Cases were 18 times more likely to have dry mouth [odds ratio 18.2, 95% confidence interval (CI) 6.7–49.6], with 54% (*n* = 27) reporting dry mouth ‘very often’ (Figure 1). There were no significant differences in the levels of oral pain, TMJ pain or dentures between groups. Only 60% of patients with inflammatory dermatological/rheumatological diagnoses brushed their teeth twice daily compared to 80% of controls (*p* = 0.037). No significant difference was seen between groups in dental appointment recency or reasons for seeking dental care at the time.

**TABLE 4:** Results from oral health questionnaire (*n* = 100)

	Cases (*n* = 50) n (%)	Controls (*n* = 50) n (%)	*p* value
Dry mouth	41 (82)	10 (20)	**0.001**
Oral pain	17 (34)	14 (28)	0.517
TMJ pain	6 (12)	1 (2)	0.05
Dentures	17 (34)	11 (22)	0.502
Tooth brushing frequency			
Twice daily	30 (60)	40 (80)	**0.037**
Once daily	16 (32)	7 (14)	
Not every day	4 (8)	3 (6)	
Time since last dentist visit			
<6 months	14 (28)	19 (38)	0.104
6–12 months	11 (22)	11 (22)	
1–4 years	13 (26)	16 (32)	
>5 years	12 (24)	4 (8)	
Reason for last dentist review			
Routine check‐up	22 (44)	21 (42)	0.454
Pain	23 (46)	21 (42)	
Treatment	1 (2)	5 (10)	
Don't know	4 (8)	3 (6)	

*Note*: The *p* values in bold signify values which reached statistical significance (<0.05).

Abbreviation: TMJ, Temporomandibular Joint.

### 4.4 Oral health examination

The dental findings from the OH examination are shown in Table 5. Cases had an average of 7.7 missing teeth compared to 4.4 in controls (*p* = 0.029). Controls had a greater number of filled teeth than cases [mean 7.6 v 5.6 respectively (*P* = 0.008)]. No significant difference was found in the number of decayed teeth or OML between the groups. Cases had a significantly greater loss of clinical attachment (a marker for periodontal disease) in all sextants compared to controls. The total LOA score is a summation of LOA in all six sextants in mm.

**TABLE 5:** Results from oral health examination (*n* = 100)

	Cases (*n* = 50)	Controls (*n* = 50)	*p* value
Decayed teeth (mean)	1.2	0.9	0.175
Filled teeth (mean)	5.6	7.6	**0.008**
Missing teeth (mean), ex 28	7.7	4.4	**0.029**
OML n (%)	10 (20)	4 (8)	0.34
Leukoplakia	1 (2)	0	
Ulceration	2 (4)	1 (2)	
Furry tongue	7 (14)	3 (6)	
LOA mean (mm)	6.9	1.9	**<0.001**

*Note*: The *p* values in bold signify values which reached statistical significance (<0.05).

Abbreviations: LOA, loss of attachment; OML, oral mucosal lesion.

### 4.5 Oral health documentation

The charts of 250 patients with severe dermatologic/rheumatologic conditions treated with systemic/biologic therapy were reviewed; 98.4% (*n* = 246) of charts did not include documentation of OH. Four charts (1.6%) documented acute dental and gingival issues.

## 5 DISCUSSION

This cross‐sectional study demonstrates that patients with severe inflammatory dermatologic and rheumatologic disease currently on systemic or biologic therapy in our departments have subjective and objective evidence of worse OH and OH‐related QoL compared to controls. Cases had significantly higher levels of self‐reported dry mouth, worse self‐reported oral hygiene, more missing teeth, and poorer periodontal health than controls. Documentation of OH in patient charts was rare.

The higher reported levels of dry mouth in the cases in this study are in keeping with previous research examining hyposalivation in rheumatologic disease.^19^ However, the prevalence of dry mouth and proportion of patients complaining of ‘very frequent’ dry mouth is high compared to previous research. Reduced oral salivary production is associated with multiple other OH problems, such as periodontitis, caries, oral candidiasis and a poorer OH‐related QoL.^19^, ^21^

Previous studies examining periodontitis in psoriasis, RA, and SS, also showed that periodontitis was more prevalent compared to controls.^13^, ^16^, ^21^ This study showed a considerable difference in LOA, a key functional marker of periodontal health, between cases and controls. The link between periodontitis and inflammatory dermatologic and rheumatologic disease may occur due to shared pathogenic features, with predisposing genetic and environmental influences, leading to immunoregulatory imbalances associated with destruction of connective and hard tissues.^27^ Periodontitis is associated with several risk factors such as unfavourable genetic polymorphisms, smoking, stress, systemic diseases and insufficient oral hygiene.^28^ Both psoriasis and periodontitis are characterised by expansion and activation of Th‐1, Th‐17 and Th‐22 T cells, with the production of associated cytokines such as interferon‐gamma, TNFα, IL‐17 AND IL‐22.^29^ Insufficient oral hygiene leads to a build‐up of dental plaque on teeth which can harden over time, forming dental calculus, which aggravates the inflammatory response of periodontal tissues.^28^ In this study, cases demonstrated significantly poorer oral hygiene habits than controls, potentially contributing to periodontal disease. Although smoking is also a risk factor for periodontal disease, the study cases had lower rates of smoking to controls.

In this study, patients with inflammatory skin or joint disease were missing almost twice as many teeth than controls. Other studies have shown an association between missing teeth and psoriasis,^13^ SS,^30^ and RA.^15^ Missing teeth have been associated with poorer OH‐related QoL.^15^

Previous studies have described an increase in OML in psoriasis,^31^, ^32^ especially fissured tongue and geographic tongue.^10^ This study recorded no cases of geographic or fissured tongue and there was no significant difference in OML frequency between the groups. This may relate to the fact that all patients with psoriasis in the study were on systemic or biologic medication, and therefore disease activity was controlled.

Strengths of this study include the detailed objective OH physical examination, which was performed by the same skilled dentist, and the use of a detailed and standardized OH questionnaire. The cohort of patients had severe inflammatory dermatologic and rheumatologic disease and were all prescribed systemic/biologic medication. The sample size was large enough to detect difference relative to controls, although the small numbers of patients with individual conditions was insufficient to analyse outcomes according to specific disease. A major limitation of this study is the wide spectrum of inflammatory dermatological and rheumatological diseases examined, meaning that there were low numbers of patients with certain conditions, such as lupus. Other limitations include the lack of additional periodontal assessment such as periodontal probing depth (the distance from the marginal gingiva to the bottom of the periodontal pocket) and bleeding on probing.^33^ These assessments were felt to be too painful and invasive to perform on patients in a busy outpatient clinic with no access to a dental chair or dental assistant. However, assessing LOA using the CPI system is designed to obtain an estimate of the lifetime accumulated destruction of the periodontal attachment and thereby permits comparisons between population groups.^1^ The fact that all cases were on systemic/biologic therapy may also have substantially reduced disease activity and may have altered OH parameters. In addition, details of disease activity and medication adherence were not recorded. Causality cannot be confirmed in this study due to the cross‐sectional design.

Future research should examine the common aetiological pathways involved in both inflammatory dermatologic/rheumatologic disease and periodontal disease. Novel methodology that objectively assesses xerostomia would also be helpful, as current best methods are subjective questioning and complex investigations such as sialometry.^34^ The sensation of dry mouth does not necessarily correlate with reduced saliva production.^35^ The objective measurement of dry mouth by sialometry has rarely been used in studies examining OH in connective tissue diseases.

Patients commencing biologic therapy for inflammatory dermatologic or rheumatologic disease are screened extensively prior to initiation of therapy to outrule certain viral and mycobacterial infections, ensure that vaccination is up to date, and educate the patient on their new medication, among other practices. The screening visit may provide an opportunity to discuss OH and refer to an OH specialist if required. This may require additional OH education for medical and nursing staff, and enhanced co‐ordination of dental and medical care. The financial implications for patients should also be considered but providing free dental reviews to patients with severe dermatologic and rheumatologic conditions may be cost‐effective.

This study shows an association between poor OH and OH‐related QoL in patients with severe inflammatory skin and joint disease treated with systemic and biologic therapy. Dermatologists should receive specific OH training to optimise the management of OH in our patients with severe inflammatory diseases.

## AUTHOR CONTRIBUTIONS

**Yvonne Kiernan:** Conceptualization (equal); Data curation (equal); Formal analysis (equal); Funding acquisition (equal); Investigation (equal); Methodology (equal); Project administration (equal); Resources (equal); Software (equal); Supervision (equal); Validation (equal); Visualization (equal); Writing – original draft (equal); Writing – review & editing (equal). **Cathal O'Connor**: Data curation (equal); Formal analysis (equal); Project administration (equal); Resources (equal); Software (equal); Validation (equal); Visualization (equal); Writing – review & editing (lead). **John Ryan:** Conceptualization (equal); Funding acquisition (equal); Investigation (equal); Methodology (equal); Project administration (equal); Resources (equal); Supervision (equal); Validation (equal); Visualization (equal); Writing – original draft (equal); Writing – review & editing (equal). **Dr. Michelle Murphy:** Conceptualization (equal); Funding acquisition (equal); Investigation (equal); Methodology (equal); Project administration (equal); Resources (equal); Supervision (equal); Validation (equal); Visualization (equal); Writing – original draft (equal); Writing – review & editing (equal).

## CONFLICT OF INTEREST

The author declares that there is no conflict of interest that could be perceived as prejudicing the impartiality of the research reported.

## ETHICS STATEMENT

The Clinical Research Ethics Committee of the Cork Teaching Hospitals approved the study protocol. Informed consent was obtained from all participating subjects prospectively and prior to invitation for the dental examination.

## Supporting information

Supporting Information S1

Supporting Information S2

## Data Availability

Data available on request.
