# Supplementary material for: Oral health in patients with severe inflammatory dermatologic and rheumatologic disease
Source: Skin Health Dis. 2022 Aug 7;3(1):e156. doi: 10.1002/ski2.156 (PMC9892474; doi:10.1002/ski2.156)
Supplement: Supplementary file 1 — Supporting Information S1 [file SKI2-3-e156-s002.pdf]

World Health Organization

Oral Health Questionnaire for Adults

**Identification number**	**Sex**		**Location**		
	Male	Female	Urban	Periurban	Rural
1. ☐☐☐☐	☐	☐	☐	☐	☐
1 4	1	2	1	2	3

2. **How old are you today?** ______________________ (Years)

3. **How many natural teeth do you have?**

No natural teeth	☐ 0
1–9 teeth	☐ 1
10–19 teeth	☐ 2
20 teeth or more	☐ 3

4. **During the past 12 months, did your teeth or mouth cause any pain or discomfort?**

Yes	☐ 1
No	☐ 2
Don't know	☐ 9
No answer	☐ 0

5. **Do you have any removable dentures?**

	Yes<br>1	No<br>2
A partial denture?	☐	☐
A full upper denture?	☐	☐
A full lower denture?	☐	☐

6. **How would you describe the state of your teeth and gums? Is it "excellent", "very good", "good", "average", "poor", or "very poor"?**

	**Teeth**	**Gums**
Excellent	☐ 1	☐ 1
Very good	☐ 2	☐ 2
Good	☐ 3	☐ 3
Average	☐ 4	☐ 4
Poor	☐ 5	☐ 5
Very poor	☐ 6	☐ 6
Don't know	☐ 9	☐ 9

7. **How often do you clean your teeth?**

Never	☐ 1
Once a month	☐ 2
2–3 times a month	☐ 3
Once a week	☐ 4
2–6 times a week	☐ 5
Once a day	☐ 6
Twice or more a day	☐ 7

8. **Do you use any of the following to clean your teeth?**
(Read each item)

	Yes 1	No 2
Toothbrush	☐	☐
Wooden toothpicks	☐	☐
Plastic toothpicks?	☐	☐
Thread (dental floss)	☐	☐
Charcoal	☐	☐
Chewstick/miswak	☐	☐
Other	☐	☐
Please specify	☐	☐

9.

	Yes	No
**a) Do you use toothpaste to clean your teeth**	☐ 1	☐ 2
**b) Do you use a toothpaste that contains fluoride?**	☐ 1	☐ 2
Don't know	☐ 9	

10. **How long is it since you last saw a dentist?**

Less than 6 months	☐ 1
6–12 months	☐ 2
More than 1 year but less than 2 years	☐ 3
2 years or more but less than 5 years	☐ 4
5 years or more	☐ 5
Never received dental care	☐ 6

11. **What was the reason of your last visit to the dentist?**

Consultation/advise	☐ 1
Pain or trouble with teeth, gums or mouth	☐ 2
Treatment/ follow-up treatment	☐ 3
Routine check-up/treatment	☐ 4
Don't know/don't remember	☐ 5

12. **Because of the state of your teeth or mouth, how often have you experienced any of the following problems during the past 12 months?**

	Very often 4	Fairly often 3	Some-times 2	No 1	Don't know 0
(a) Difficulty in biting foods	☐	☐	☐	☐	☐
(b) Difficulty chewing foods	☐	☐	☐	☐	☐
(c) Difficulty with speech/trouble pronouncing words	☐	☐	☐	☐	☐
(d) Dry mouth	☐	☐	☐	☐	☐
(e) Felt embarrassed due to appearance of teeth	☐	☐	☐	☐	☐
(f) Felt tense because of problems with teeth or mouth	☐	☐	☐	☐	☐
(g) Have avoided smiling because of teeth	☐	☐	☐	☐	☐
(h) Had sleep that is often interrupted	☐	☐	☐	☐	☐
(i) Have taken days off work	☐	☐	☐	☐	☐
(j) Difficulty doing usual activities	☐	☐	☐	☐	☐
(k) Felt less tolerant of spouse or people who are close to you	☐	☐	☐	☐	☐
(l) Have reduced participation in social activities	☐	☐	☐	☐	☐

13. **How often do you eat or drink any of the following foods, even in small quantities?**
(Read each item)

	Several times a day 6	Every day 5	Several times a week 4	Once a week 3	Several times a month 2	Seldom /never 1
Fresh fruit	☐	☐	☐	☐	☐	☐
Biscuits, cakes, cream cakes	☐	☐	☐	☐	☐	☐
Sweet pies, buns	☐	☐	☐	☐	☐	☐
Jam or honey	☐	☐	☐	☐	☐	☐
Chewing gum containing sugar	☐	☐	☐	☐	☐	☐
Sweets/candy	☐	☐	☐	☐	☐	☐

Lemonade, Coca Cola or other soft drinks ..	☐	☐	☐	☐	☐	☐
Tea with sugar	☐	☐	☐	☐	☐	☐
Coffee with sugar	☐	☐	☐	☐	☐	☐

(Insert country-specific items)

14. **How often do you use any of the following types of tobacco?**
(Read each item)

	Every day	Several times a week	Once a week	Several times a month	Seldom	Never
	6	5	4	3	2	1
Cigarettes	☐	☐	☐	☐	☐	☐
Cigars	☐	☐	☐	☐	☐	☐
A pipe	☐	☐	☐	☐	☐	☐
Chewing tobacco	☐	☐	☐	☐	☐	☐
Use snuff	☐	☐	☐	☐	☐	☐
Other	☐	☐	☐	☐	☐	☐

Please specify ____________________

15. **During the past 30 days, on the days you drank alcohol, how many drinks did you *usually drink per day*?**

Less than 1 drink .... ☐ 0
1 drink .... ☐ 1
2 drinks .... ☐ 2
3 drinks .... ☐ 3
4 drinks .... ☐ 4
5 or more drinks .... ☐ 5
Did not drink alcohol during the past 30 days .... ☐ 9

16. **What level of education have you completed?**

No formal schooling .... ☐ 1
Less than primary school .... ☐ 2
Primary school completed .... ☐ 3
Secondary school completed .... ☐ 4
High school completed .... ☐ 5
College/university completed .... ☐ 6
Postgraduate degree .... ☐ 7

(Insert country-specific categories)

That completes our questionnaire
Thank you very much for your cooperation!

Year	Month	Day	Interviewer	District	Country
☐☐	☐☐	☐☐	☐☐	☐☐	☐☐☐☐
