# Supplementary material for: Oral health in patients with severe inflammatory dermatologic and rheumatologic disease
Source: Skin Health Dis. 2022 Aug 7;3(1):e156. doi: 10.1002/ski2.156 (PMC9892474; doi:10.1002/ski2.156)
Supplement: Supplementary file 2 — Supporting Information S2 [file SKI2-3-e156-s001.pdf]

World Health Organization

World Health Organization
Oral Health Assessment Form for Adults, 2013

Leave blank (1) ☐☐☐☐ (4) | Year / Month / Day (5) ☐☐☐☐☐☐ (10) | Identification No. (11) ☐☐☐☐ (14) | Orig/Dupl ☐ (15) | Examiner (16) ☐☐ (17)

General information: **Sex** 1=M, 2=F ☐ (18) **Date of birth** (19) ☐☐☐☐☐☐ (24) **Age in years** (25) ☐☐ (26)

____
(Name)

Ethnic group (27) ☐☐ (28) **Other group**(29) ☐☐ (30) **Years in school** (31) ☐☐ (32) **Occupation** ☐ (33)

Community (geographical location) (34) ☐☐ (35) **Location** Urban (1) Periurban (2) Rural (3) ☐ (36)

Other data ____________________ (37) ☐☐ (38) **Other data** ____________________ (39) ☐☐ (40)

Other data ____________________ (41) ☐☐ (42) **Extra-oral examination** __________ (43) ☐☐ (44)

Dentition status

	18	17	16	15	14	13	12	11	21	22	23	24	25	26	27	28	
Crown (45)	☐	☐	☐	☐	☐	☐	☐	☐	☐	☐	☐	☐	☐	☐	☐	☐	60)
Root (61)	☐	☐	☐	☐	☐	☐	☐	☐	☐	☐	☐	☐	☐	☐	☐	☐	(76)
Crown (77)	☐	☐	☐	☐	☐	☐	☐	☐	☐	☐	☐	☐	☐	☐	☐	☐	(92)
Root (93)	☐	☐	☐	☐	☐	☐	☐	☐	☐	☐	☐	☐	☐	☐	☐	☐	(108)
	48	47	46	45	44	43	42	41	31	32	33	34	35	36	37	38	

Permanent teeth

Status

0 = Sound
1 = Caries
2 = Filled w/caries
3 = Filled, no caries
4 = Missing due to caries
5 = Missing for any another reason
6 = Fissure sealant
7 = Fixed dental prosthesis/crown abutment, veneer, implant
8 = Unerupted
9 = Not recorded

Periodontal status (CPI Modified)

	18	17	16	15	14	13	12	11	21	22	23	24	25	26	27	28	
Bleeding (109)	☐	☐	☐	☐	☐	☐	☐	☐	☐	☐	☐	☐	☐	☐	☐	☐	(124)
Pocket (125)	☐	☐	☐	☐	☐	☐	☐	☐	☐	☐	☐	☐	☐	☐	☐	☐	(140)
Bleeding (141)	☐	☐	☐	☐	☐	☐	☐	☐	☐	☐	☐	☐	☐	☐	☐	☐	(156)
Pocket (157)	☐	☐	☐	☐	☐	☐	☐	☐	☐	☐	☐	☐	☐	☐	☐	☐	(172)
	48	47	46	45	44	43	42	41	31	32	33	34	35	36	37	38	

Gingival bleeding

Score
0 = Absence of condition
1 = Presence of condition
9 = Tooth excluded
X = Tooth not present

Pocket

Score
0 = Absence of condition
1 = Pocket 4–5 mm
2 = Pocket 6 mm or more
9 = Tooth excluded
X = Tooth not present

World Health Organization
Oral Health Assessment Form for Adults, 2013

Loss of attachment

Severity
0 = 0–3 mm
1 = 4–5 mm Cemento-enamel junction (CEJ) within black band
2 = 6–8 mm CEJ between upper limit of black band and 8.5 mm ring
3 = 9–11 mm CEJ between 8.5 mm and 11.5 mm ring
4 = 12 mm or more CEJ beyond 11.5 mm ring
X = Excluded sextant
9 = Not recorded

* Not recorded under 15 years of age

Index teeth

	17/16	11	26/27	
(173)	☐	☐	☐	(175)
(176)	☐	☐	☐	(178)
	47/46	31	36/37	

Enamel fluorosis ☐ (179)

Severity
0 = Normal
1 = Questionable
2 = Very mild
3 = Mild
4 = Moderate
5 = Severe
8 = Excluded (crown, restoration, "bracket")
9 = Not recorded (unerupted tooth)

Dental erosion

Severity ☐ (180)

0 = No sign of erosion
1 = Enamel lesion
2 = Dentinal lesion
3 = Pulp involvement

Number of teeth affected

(181) ☐☐ (182)

Dental trauma

Status ☐ (183)

0 = No sign of injury
1 = Treated injury
2 = Enamel fracture only
3 = Enamel and dentine fracture
4 = Pulp involvement
5 = Missing tooth due to trauma
6 = Other damage
9 = Excluded tooth

Number of teeth affected

(184) ☐☐ (185)

Oral mucosal lesions

☐ (186)
☐ (187)
☐ (188)

☐ (189)
☐ (190)
☐ (191)

Condition

0 = No abnormal condition
1 = Malignant tumour (oral cancer)
2 = Leukoplakia
3 = Lichen planus
4 = Ulceration (aphthous, herpetic, traumatic)
5 = Acute necrotizing ulcerative gingivitis (ANUG)
6 = Candidiasis
7 = Abscess
8 = Other condition (specify if possible)
9 = Not recorded

Location

0 = Vermillion border
1 = Commissures
2 = Lips
3 = Sulci
4 = Buccal mucosa
5 = Floor of the mouth
6 = Tongue
7 = Hard and/or soft palate
8 = Alveolar ridges/gingiva
9 = Not recorded

Denture(s)

Upper ☐ (192) **Lower** ☐ (193)

Status

0 = No denture
1 = Partial denture
2 = Complete denture
9 = Not recorded

Intervention urgency ☐ (194)
0 = No treatment needed
1 = Preventive or routine treatment needed
2 = Prompt treatment (including scaling) needed
3 = Immediate (urgent) treatment needed due to pain or infection of dental and/or oral origin
4 = Referred for comprehensive evaluation or medical/dental treatment (systemic condition)
